# Stressing the Regulatory Role of Long Non-Coding RNA in the Cellular Stress Response during Cancer Progression and Therapy

**DOI:** 10.3390/biomedicines10051212

**Published:** 2022-05-23

**Authors:** Yi-Zhen Wu, Yong-Han Su, Ching-Ying Kuo

**Affiliations:** 1 Department of Clinical Laboratory Sciences and Medical Biotechnology, College of Medicine, National Taiwan University, Taipei 100229, Taiwan; b03404004@ntu.edu.tw (Y.-Z.W.); b04404041@ntu.edu.tw (Y.-H.S.); 2 Department of Laboratory Medicine, National Taiwan University Hospital, Taipei 100225, Taiwan

**Keywords:** long non-coding RNA, cellular stress, therapeutic-induced stress, stress response, therapy resistance

## Abstract

Cellular stress response is an important adaptive mechanism for regulating cell fate decision when cells confront with stress. During tumorigenesis, tumor progression and the course of treatment, cellular stress signaling can activate subsequent response to deal with stress. Therefore, cellular stress response has impacts on the fate of tumor cells and tumor responsiveness relative to therapeutic agents. In recent years, attention has been drawn to long non-coding RNAs (lncRNAs), a novel class of RNA molecules with more than 200 nucleotides in length, which has little protein-coding potential and possesses various functions in multiple biological processes. Accumulating evidence has shown that lncRNAs are also engaged in the regulation of cellular stress response, particularly in cancers. Here, we summarize lncRNAs that have been reported in the adaptive response to major types of cellular stress including genotoxic, hypoxic, oxidative, metabolic and endoplasmic reticulum stress, all of which are often encountered by cancer cells. Specifically, the molecular mechanisms of how lncRNAs regulate cellular stress response during tumor progression or the development of therapy resistance are emphasized. The potential clinical applications of stress-responsive lncRNAs as biomarkers will also be discussed.

## 1. Introduction

During tumorigenesis and tumor progression, cancer cells require a sufficient supply of nutrients, oxygen and growth factor stimulation to support their rapid growth. Nevertheless, the uncontrolled proliferation, confined living space and poor vascularization of solid tumor often restrict the continuous supply of nutrients and oxygen in the tumor microenvironment, generating different types of cellular stress including hypoxic stress, oxidative stress, endoplasmic reticulum (ER) stress and metabolic stress [1,2]. Moreover, dysfunctional DNA repair pathways in cancer cells lead to the accumulation of DNA damages and the occurrence of genotoxic stress [3]. Cancer cells have evolved a variety of cellular mechanisms to confront different types of stress. Currently, plenty of therapeutic agents have been developed for treating cancer, and some of them are stress-inducing agents that trigger cancer cell death by provoking stress [4,5]. When encountering these stimulations, type-specific stress-responsive pathways are activated to overcome stresses and may further confer therapeutic resistance in cancer [6,7]. Various molecules including long non-coding RNAs (lncRNAs) participate in these stress-response pathways.

LncRNAs is a group of RNA transcripts longer than 200 nucleotides with little protein-coding capacity. They compose of a large proportion of human transcriptome and have been demonstrated to regulate many biological processes and the development of human diseases, such as cancers or cardiovascular diseases [8,9]. LncRNAs share similar processes of biogenesis with mRNAs. They are mainly transcribed by RNA polymerase II (Pol II) and possess 5′ methyl guanosine cap and 3′ poly (A) tail [10]. According to their genomic location, lncRNAs are classified into five categories: (1) sense—overlaps with the exons of the protein-coding gene on the same strand; (2) anti-sense—located on the anti-sense of the protein-coding gene; (3) intronic—located entirely within the intron of the protein-coding gene; (4) intergenic—locates in the intergenic region of two protein-coding genes and is transcribed in the same orientation; (5) bidirectional—located within 1 kb of the promoter of the protein-coding gene and is transcribed in the opposite direction [11,12]. Owing to the diversity of structure, sequence and subcellular localization, lncRNAs are able to collaborate with various molecules to regulate a wide variety of biological pathways in different cellular compartments [10]. For example, lncRNAs regulate gene expression at multiple levels. Nuclear lncRNAs recruit DNA methyltransferase to promote DNA methylation for regulating epigenetics [13]. LncRNAs are also demonstrated to interact with splicing factors to participate in alternative splicing [14]. In cytoplasm, lncRNAs can act as competing endogenous RNAs (ceRNAs) to sponge microRNAs (miRNAs) and prevent miRNA-mediated mRNA degradation [15]. Over the past few years, lncRNAs have been demonstrated to be involved in the regulation of cancer initiation, progression and therapy resistance in diverse ways [16,17,18,19]. For instance, lncRNAs are reported to regulate the stemness and epithelial–mesenchymal transition (EMT) of cancer cells, which contributes to the progression or chemotherapeutic resistance of cancers [18]. LncRNAs participate in the regulation of critical signaling pathways, such as p53, AKT or Notch signaling pathways and can further affect the development of cancers [20]. In addition, the occurrence of metabolic reprogramming, a hallmark of cancer, also requires the involvement of lncRNAs [19]. As a result, the multifaceted function of lncRNAs renders them as potential therapeutic targets or biomarkers in multiple cancers [21]. Albeit lncRNAs have been well identified in regulating diverse cellular processes, the role of lncRNAs in stress response signaling is relatively unclear. Emerging studies have started to recognize the existence and the function of stress-responsive and stress-regulating lncRNAs. These lncRNAs are either induced or inhibited upon stress stimulation to participate in the stress response, or they can directly affect the occurrence of stress. In this review, we mainly focus on the regulatory role of the stress-responsive lncRNAs under major stressed conditions. The involvement of these lncRNAs in the stress response not only modulates cancer cell fate but also impacts on therapeutic efficacy, shedding light on the development of new therapeutic strategies or novel biomarkers for cancer.

## 2. Genotoxic Stress

Genomic instability in cancer cells is associated with the accumulation of endogenous DNA damages or the exposure to exogenous genotoxic agents and may further promote cancer progression. A comprehensive process denoted DNA damage response (DDR) is activated to resolve the genotoxic stress. DDR is composed of DNA lesion sensing, transducing damaging signals and transcriptionally activating cellular processes including cell cycle arrest, DNA repair, senescence and apoptosis. Several types of DNA damage have been reported, and DNA double-strand breaks (DSBs) are the most lethal for cancer cells. To overcome these damages, non-homologous end joining (NHEJ) and homologous recombination (HR) are the most dominant mechanisms that repair the lesions on the helix backbone. The repair process is exerted by different components involved in DDR machinery, including the sensors (MRN complex and 9-1-1 complex), the kinases (ATM, ATR and CHK1/2) and the DNA repair mediators (γH2AX, 53BP1, RAD51 and BRCA1), which are all indispensable in maintaining genomic integrity [7,22,23]. In addition, the leading transcriptional regulator in DDR response, p53, participates in multiple cellular processes upon DNA damage. p53 not only suppresses cell growth but also enhances apoptosis and transcriptionally activates the components involved in DNA repair processes. [24,25].

**Figure 1:**
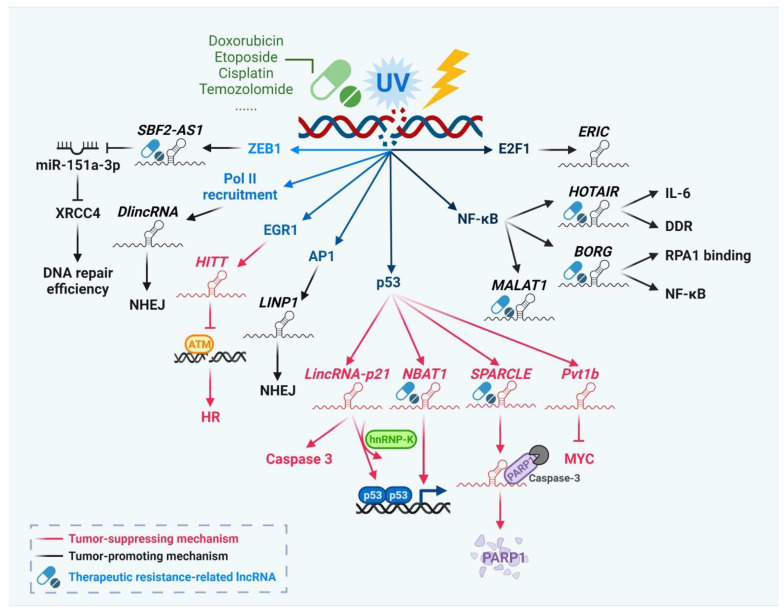
LncRNAs play multiple roles in DNA damage response (DDR) to address genotoxic stress. Under exogenous genotoxic agent-mediated DNA damage, lncRNAs are involved in a variety of regulatory roles of DDR and modulate cell fates. The key upstream transcription factors of each distinct lncRNA and the downstream targeted mechanisms or the related cellular processes are summarized. LncRNAs that particularly impact therapeutic susceptibility are marked. AP1 (activator protein 1), ATM (ataxia telangiectasia mutated), *BORG* (BMP/OP-responsive gene), *DlincRNA* (damage-induced long non-coding RNA), E2F1 (E2F transcription factor 1), EGR1 (early growth response protein 1), *ERIC* (E2F1-regulated inhibitor of cell death), *HITT* (HIF-1α inhibitor at translation level), hnRNP-K (heterogeneous nuclear ribonucleoprotein K), *HOTAIR* (HOX transcript antisense RNA), HR (homologous recombination), IL-6 (interleukin 6), *LINP1* (LncRNA In non-homologous end joining pathway 1), *MALAT1* (metastasis associated lung adenocarcinoma transcript 1), *NBAT1* (neuroblastoma associated transcript 1), NF-κB (nuclear factor-κB), NHEJ (non-homologous end joining), PARP1 (poly (ADP-ribose) polymerase 1), Pol II (RNA polymerase II), *Pvt1b* (plasmacytoma variant 1b), RPA1 (replication protein A1), *SBF2-AS1* (SBF2 antisense RNA 1), *SPARCLE* (suicidal PARP-1 cleavage enhancer), XRCC4 (X-ray repair cross complementing 4) and ZEB1 (zinc finger E-box binding homeobox 1). This figure was created with BioRender.com.

LncRNAs have been reported to play a critical role in gating genomic stability upon DNA damage. After being transcriptionally regulated by specific molecules, lncRNAs link different biological processes or collaborate with other regulators in DDR [26,27,28] (Figure 1). Several lncRNAs are directly induced by the binding of p53 at their promoter regions and promote apoptosis or gene suppression in a p53-dependent manner [29,30,31]. The p53-induced lncRNAs activate apoptosis by binding to PARP1 or downregulating cell proliferation through the inhibition of MYC [32,33]. In addition to p53, lncRNAs are regulated by other DDR-mediated transcription factors. *ERIC* is transcriptionally activated by E2F1 following DNA damage and further inhibits the DNA damage-induced apoptosis in a p53-independent manner [34]. Moreover, NF-κB directly induces *HOTAIR* under the genotoxic stress, and elevated *HOTAIR* promotes DDR, cellular senescence, IL-6 secretion and sustains NF-κB activation [35].

LncRNAs impact the distinct DSB repair process upon DNA damage [26,27]. *DlinRNAs* are transcribed in the bidirectional forms after the MRN complex-mediated recruitment of RNA Pol II, and they further act as the precursors of small non-coding RNA *DDRNAs* to facilitate the recruitment of DNA repair proteins such as 53BP1 to the DSB sites [36]. *LINP1* has been reported to strengthen the binding efficiency of Ku70/80 complex and DNA-PKcs to the break sites to promote NHEJ after being activated by AP1 upon the formation of DSBs [37]. Furthermore, the HR repair pathway can also be regulated by lncRNAs. *HITT,* which is transcriptionally activated by early growth response 1 (EGR1), inhibits the recruitment of ATM to the damage sites and further deregulates the HR repair signaling [38].

The development of anti-cancer treatments with genotoxic agents such as ionizing radiation and chemotherapeutics is based on the inability of cancer cells to efficiently repair DNA damages. However, cancer cells with intrinsic or acquired resistance to DNA damage-induced cytotoxicity are less sensitive to genotoxic stress, rendering poor clinical outcomes in patients undergone genotoxic therapies [27,39]. Evidence has piled up to show that lncRNAs play a role in the resistance to genotoxic therapies. *BORG* is highly responsive to multiple chemotherapeutic agents such as doxorubicin or 5-fluorouracil (5-FU) and leads to chemoresistance by sustaining the activation of NF-κB [40]. *MALAT1* is co-activated by NF-κB and p53 following the treatment of temozolomide (TMZ) in glioblastoma (GBM) and promotes resistance to TMZ [41]. *SBF2-AS1* is activated by zinc finger E-box binding homeobox 1 (ZEB1) and regulates the XRCC4-mediated DSB repair machinery by sponging miR-151a-3p. This protective effect is amplified by exosomal *SBF-AS1* secretion to influence the neighboring GBM cells [42]. Moreover, lncRNAs can control cell fate depending on the degree of stress. *Nrf2-lncRNA* is induced by p53 upon weak DNA damage and promotes the expression of itself and Plk2/p21^cip1^ by competing with miR-128 and miR-224, further leading to the upregulation of nuclear factor erythroid 2-related factor 2 (NRF2) and cell growth. In contrast, stronger DNA damage dampens the p53-mediated transcriptional enhancement of *Nrf2-lncRNA* [43].

## 3. Hypoxic Stress

Hypoxia is defined as a reduction in oxygen supply from vessels and accompanies several pathological states including cancer. As a hallmark of tumor microenvironment, hypoxia modulates multiple aspects of tumor progression, such as promoting survival signaling response, metastasis, anti-apoptosis, central metabolic reprogramming and further impacts therapeutic outcomes. Under hypoxic stress, various transcription factors are upregulated to activate downstream cellular signals. Hypoxia-inducible-factor-1 (HIF-1) complex is one of the major mediators for regulating oxygen-dependent transcriptional responses. The activation of HIF-1αregulates multiple biological pathways, including glycolysis, metastasis and cell immortalization [44,45,46,47].

**Figure 2:**
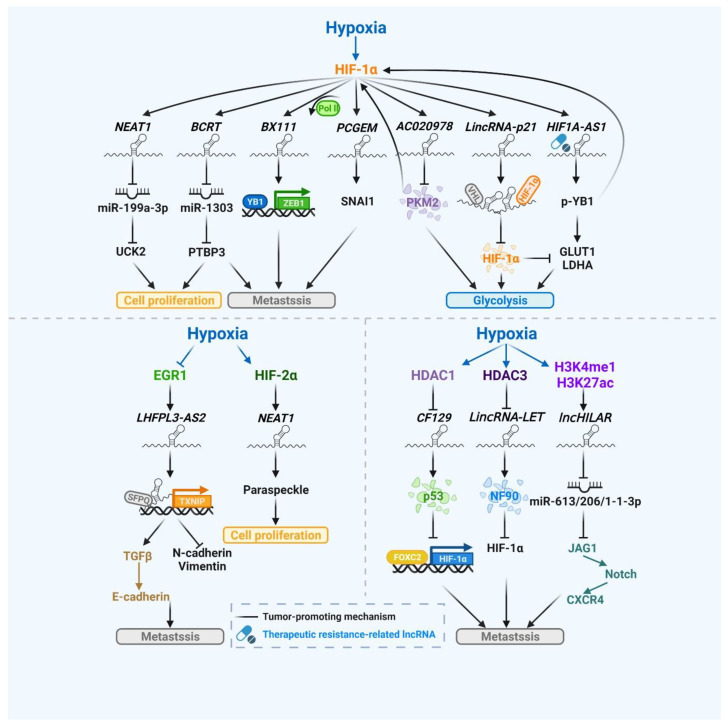
LncRNAs involved in the hypoxic stress response are regulated through both transcriptional control and epigenetic modification. Upon hypoxia, lncRNAs are regulated by not only specific transcription factors such as HIF-1α, HIF-2α and EGR1 but also hypoxia-dependent epigenetic modification. The regulated lncRNAs further turn on the tumor-promoting processes, including cell proliferation, metastasis and glycolysis to overcome the hypoxic stress. *BCRT* (breast cancer-related transcript 1), CXCR4 (C-X-C motif chemokine receptor 4), FOXC2 (forkhead box C2), GLUT1 (glucose transporter 1), HDAC1/3 (histone deacetylase 1/3), HIF-1α (hypoxia-inducible factor 1-alpha), *HIF1A-AS1* (HIF1A antisense RNA 1), HIF-2α (Hypoxia-inducible factor 2-alpha), *lncHILAR* (hypoxia-induced long non-coding RNA), JAG1 (Jagged1), LDHA (lactate dehydrogenase A), *LincRNA-LET* (lncRNA low expression in tumor), *LHFPL3-AS1* (lipoma HMGIC fusion partner-like 3 antisense RNA 1), *NEAT1* (nuclear paraspeckle assembly transcript 1), NF90 (nuclear factor 90), *PCGEM* (prostate cancer gene expression marker 1), PKM2 (pyruvate kinase M2), PTBP3 (polypyrimidine tract binding protein 3), SFPQ (splicing factor proline and glutamine rich), SNAI1 (snail family transcriptional repressor 1), TGF-β (transforming growth factor beta), TXNIP (thioredoxin interacting protein), UCK2 (uridine-cytidine kinase 2), VHL (Von Hippel–Lindau tumor suppressor) and YB1 (Y-box protein 1). This figure was created with BioRender.com.

In addition to the largely revealed transcriptomes of protein-coding genes in response to hypoxic tumor microenvironments, accumulating evidence also suggests the involvement of lncRNAs under hypoxia [48,49] (Figure 2). For example, HIF-1α induces specific lncRNAs such as *PCGEM1* or *BX111* by directly binding or recruiting Pol II to the hypoxia-responsive element (HRE) at the promoter of target lncRNAs. The upregulated lncRNAs further enhance molecules involved in the EMT to promote migration and invasion of cancer cells [50,51]. HIF-1α has been demonstrated as a key mediator of aerobic glycolysis under hypoxia [52] and studies have shown that lncRNAs also participate in this process. Upon the induction by HIF-1α, lncRNAs can translocate to the cytoplasm for diverse functions. *AC020978* directly interacts with and stabilizes pyruvate kinase isozymes M2 (PKM2) to promote PKM2 nuclear translocation and further facilitates PKM2-enhanced HIF-1α transcriptional activity [53]. *LincRNA-p21* or *HIF1A-AS1* can provide positive feedback on the expression of HIF-1α by preventing VHL-mediated ubiquitylation or by phosphorylating the RNA-binding protein to promote the translation of HIF-1α, respectively [54,55]. These reciprocal feedback loops between HIF-1α and the target lncRNAs further upregulate both lncRNAs and the glycolytic genes such as *GLUT1*, *LDHA* and *PKM2* to promote glycolysis. Moreover, lncRNAs have been reported to function as ceRNAs to regulate target genes by modulating the expression or the activity of miRNAs [15,56,57]. In hypoxic hepatocellular carcinoma (HCC), *NEAT1* sponges miR-199a-3p to promote cell proliferation by increasing the expression of *UCK2* [58]. In addition, after the transcriptional activation by HIF-1α in breast cancer (BC), *BCRT* acts as a miRNA sponge to inhibit miR-1303. The reduced level of miR-1303 prevents *PTBP3* from degradation and further promotes cell proliferation and metastasis [59].

It has been reported that the expression of specific lncRNAs can be modulated by DNA methylation at their promoter regions [60,61]; furthermore, the hypoxic status can induce abnormal DNA methylation and acetylation, which indicates that HIF-1α not only directly binds to the promoter regions of target lncRNAs but also modulates lncRNAs with epigenetic modifications [48]. In renal cell carcinoma, *lncHILAR* expression is upregulated via H3K4me1 and H3K27ac enrichments at the promoter region of *lncHILAR*. In addition, the elevated *lncHILAR* acts as the ceRNA for miR-613/206/1-13p and further activates the Jagged1/Notch/CXCR4 signaling pathway to promote metastasis under hypoxia [62]. On the other hand, tumor-suppressive lncRNAs such as *CF129* or *LET* are inhibited by HIF-1α-mediated histone deacetylase (HDAC) recruitment. HIF-1α suppresses lncRNA expression by promoting HDAC1 or HDAC3 recruitment to promoter regions. The downregulated lncRNAs stabilize downstream targets by inhibiting proteins from proteasomal degradation and result in a positive feedback loop to enhance the HIF-1α response [63,64].

In addition to the major role of HIF-1α in regulating hypoxic stress signaling, other hypoxia-responsive transcription factors also play important roles in the hypoxic response [47]. In BC, *NEAT1* is transcriptionally upregulated by HIF-2α under hypoxia and regulates the nuclear structure, further contributing to BC cell proliferation [65]. *LHFPL-AS2*, a tumor-suppressive lncRNA in lung cancer, is downregulated under hypoxia due to the reduction in EGR1 expression, resulting in the transcriptional repression of thioredoxin-interacting protein (*TXNIP)* and promoting tumor metastasis [66].

## 4. Oxidative Stress

**Figure 3:**
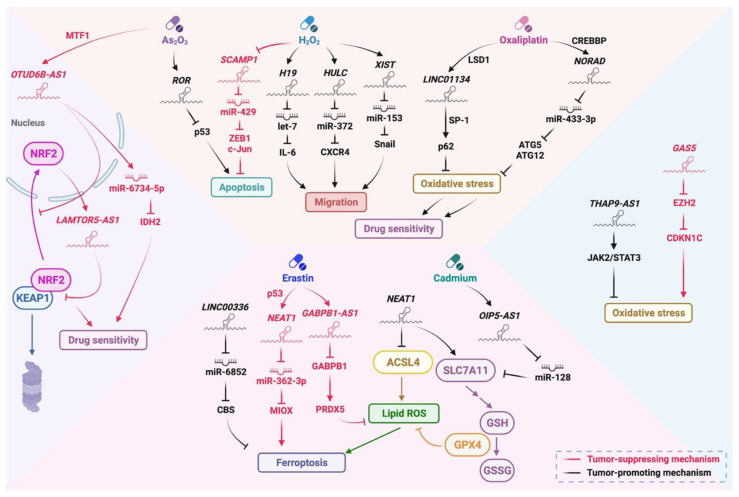
LncRNAs participate in the regulation of oxidative stress response in multiple aspects. LncRNAs such as *OTUD6B-AS1* and *LAMTOR5-AS1* regulate the function of NRF2 and further affect drug sensitivity. *GAS5* and *THAP9-AS1* are reported to induce and reduce oxidative stress, respectively. On the other hand, lncRNAs are demonstrated to be modulated by oxidative stress-inducing chemotherapeutic agents, including As_2_O_3_, H_2_O_2_, oxaliplatin, erastin and cadmium, to manage therapeutic efficacy through a series of biological processes, such as apoptosis, migration and ferroptosis. NRF2 (nuclear factor erythroid 2-related factor 2), KEAP1 (kelch-like ECH-associated protein 1), MTF1 (metal regulatory transcription factor 1), IDH2 (isocitrate dehydrogenase 2), ZEB1 (zinc finger E-box binding homeobox 1), IL-6 (interleukin 6), CXCR4 (C-X-C motif chemokine receptor 4), LSD1 (Lysine-specific demethylase 1), SP-1 (specificity protein 1), CREBBP (CREB binding protein), ATG5 (autophagy related 5), ATG12 (autophagy related 12), JAK2 (janus kinase 2), STAT3 (signal transducer and activator of transcription 3), EZH2 (enhancer of zeste homolog 2), CDKN1C (cyclin dependent kinase inhibitor 1C), CBS (cystathionine beta-synthase), MIOX (myo-inositol oxygenase), GABPB1 (GA binding protein transcription factor subunit beta 1), PRDX5 (peroxiredoxin 5), ACSL4 (acyl-CoA synthetase long chain family member 4), SLC7A11 (solute carrier family 7 member 11), GSH (glutathione), GSSG (glutathione disulfide) and GPX4 (glutathione peroxidase 4). This figure was created with BioRender.com.

Oxidative stress is initiated when oxidants and the antioxidants are imbalanced. Intracellular oxidants, including reactive oxygen species (ROS), reactive nitrogen species (RNS) and lipid peroxides, are often higher in tumor cells than in normal cells. These oxidants are generated by aberrant metabolisms or after exposure to different stimuli, such as radiation, hypoxia or chemotherapeutic drugs, and the accumulation of oxidants can induce DNA damage, comprise cell growth or further result in the tumor cell death [67]. To survive from excessive oxidative stress, tumor cells often activate different antioxidant transcription factors and signaling pathways to initiate oxidative stress response [68], which has been demonstrated to require the participation of lncRNAs in multiple aspects (Figure 3).

NRF2, one of the most important antioxidant transcription factors, binds to antioxidant response element (ARE) to regulate a series of antioxidant genes, which is responsible for producing antioxidants such as glutathione (GSH) or thioredoxin [69]. Some lncRNAs are reported to be involved in the NRF2-regulated signaling pathway. For example, lncRNA *LAMTOR5-AS1* is upregulated by NRF2 in chemo-sensitive cells. *LAMTOR5-AS1* regulates the interaction between NRF2 and Kelch-like ECH-associated protein 1 (KEAP1) to inhibit NRF2 transcriptional activity under chemotherapeutic drugs treatment, which finally results in an increase in drug sensitivity in osteosarcoma [70]. LncRNA *OTUD6B-AS1*, which is induced by metal-regulatory transcription factor 1 (MTF1) after the treatment of arsenic trioxide (As_2_O_3_), retains NRF2 in the cytoplasm to inhibit its function. On the other hand, *OTUD6B-AS1* improves the stability of miR-6734-5p, a miRNA that targets isocitrate dehydrogenase 2 (IDH2) through direct binding to decrease the expression of *IDH2*. The inhibition of NRF2 function and IDH2 expression leads to the accumulation of oxidative stress and the cytotoxicity under As_2_O_3_ treatment in bladder cancer [71].

In addition to the NRF2 pathway, lncRNAs can also regulate oxidative stress response through multiple signaling pathways and affect different biological processes, such as apoptosis, autophagy or inflammatory pathways. LncRNA *H19* and *HULC* are found to activate inflammatory responses through miRNA sponging under oxidative stress. *H19* and *HULC* are upregulated under H_2_O_2_ and glucose oxidase treatment. *H19* sponges let-7 to increase IL-6 expression and *HULC* sponges miR-372 to upregulate CXCR4, which promotes the migratory ability of cholangiocarcinoma cells [72]. LncRNA *XIST* is shown to be upregulated for promoting the migratory ability of osteosarcoma cells under H_2_O_2_ treatment. Mechanistically, *XIST* sponges miR-153 to increase the expression of Snail and further promotes migration [73]. As_2_O_3_-induced oxidative stress upregulates the expression of lncRNA *ROR*, which reduces p53 expression to prevent apoptosis [74]. H_2_O_2_-induced oxidative stress downregulates lncRNA *SCAMP1* to enhance the expression of miR-429. miR-429 then targets ZEB1 and c-Jun for degradation and promotes autophagy, which eventually contributes to the increase in apoptosis and the decrease in tumorigenesis [75]. Oxaliplatin is a chemotherapeutic drug that can arouse oxidative stress in cells [76]. In oxaliplatin-resistant HCC cells, the expression of lncRNA *LINC01134* is increased by lysine-specific demethylase 1 (LSD1)-mediated demethylation. *LINC01134* recruits SP-1 to the *SQSTM1*/p62 promoter for enhancing its expression, which improves mitochondrial function to reduce ROS production and finally results in the resistance to oxaliplatin [77]. LncRNA *NORAD* is also reported to contribute to oxaliplatin resistance. Oxaliplatin promotes CREBBP-mediated H3K27ac on *NORAD* promoters to upregulate *NORAD* expression. *NORAD* then sponges miR-433-3p to increase the expression of autophagy-related genes *ATG5* and *ATG12*, which helps in alleviating DNA damage and oxidative stress and eventually confers oxaliplatin resistance in gastric cancer (GC) cells [78]. Some lncRNAs directly regulate the accumulation of endogenous oxidative stress. LncRNA *THAP9-AS1* is highly expressed in osteosarcoma tissues and increases methylation at the promoter region of the suppressor of cytokine signaling 3 (SOCS3), which activates JAK2/STAT3 signaling to reduce oxidative stress [79]. Conversely, lncRNA *GAS5* promotes the accumulation of oxidative stress and apoptosis to inhibit the growth of melanoma cells. Mechanistically, *GAS5* binds E2F4 to prevent the binding of E2F4 on the *EZH2* promoter region, causing a decrease in *EZH2* expression and EZH2-mediated *CDKN1C* downregulation. This finally leads to the enhancement of oxidative stress and apoptotic cell death [80].

Ferroptosis, a new type of regulated cell death induced by oxidative stress, has been recently implicated to regulate tumor initiation or progression [81]. It is mainly initiated by the accumulation of iron-mediated ROS or lipid peroxides [82] and is shown to be regulated by lncRNAs in the following studies. Erastin, a ferroptosis inducer, upregulates lncRNA *NEAT1* through p53 in HCC cells. *NEAT1* sponges miR-362-3p to increase myo-inositol oxygenase (MIOX) expression and promotes ferroptosis, which is shown to reverse erastin resistance in HCC [83]. On the other hand, *NEAT1* inhibits erastin-induced ferroptosis by not only increasing GPX4 and SLC7A11-mediated antioxidative pathways but also decreasing ACSL4-mediated lipid peroxidation, which may confer the resistance to erastin in non-small cell lung cancer (NSCLC) cells [84]. LncRNA *GABPB1-AS1* is upregulated to bind its sense mRNA, *GABPB1*, under erastin treatment, and this results in the decrease in *GABPB1* translation. GABPB1 downregulation inhibits peroxiredoxin-5 (PRDX5) expression to abrogate antioxidative capacities, which finally increases the accumulation of lipid ROS and promotes ferroptosis in HCC cells [85]. LncRNA *LINC00336* is upregulated through ELAVL1-mediated RNA stabilization. *LINC00336* sponges miR-6852 to enhance cystathionine-β-synthase (CBS) expression, which produces more antioxidant glutathione to alleviate oxidative stress and inhibit ferroptosis [86]. Cadmium (Cd) is found to suppress ferroptosis for promoting prostate tumor growth by upregulating lncRNA *OIP5-AS1*. *OIP5-AS1* sponges miR-128 to increase the expression of miR-128-targeted SLC7A11, which is involved in the production of glutathione. Therefore, *OIP5-AS1* is able to reduce Cd-induced ferroptosis through the miR-128/SLC7A11 axis [87].

## 5. Metabolic Stress

**Figure 4:**
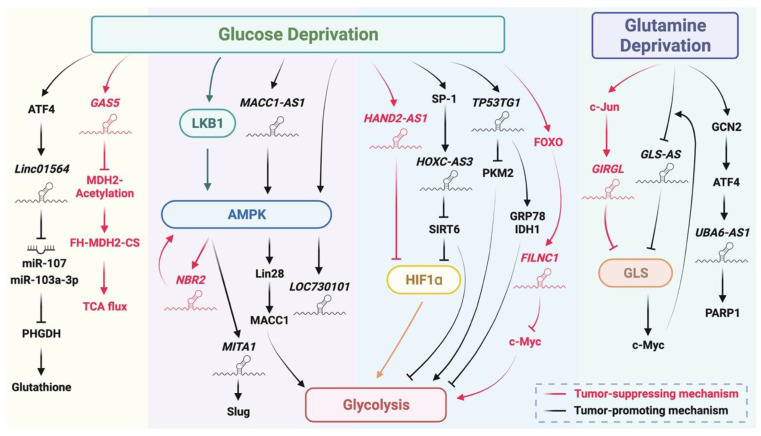
Metabolic stress-responsive lncRNAs are involved in the regulation of cell fate decision under glucose or glutamine deprivation. Glucose starvation induces lncRNA *NBR2*, *MITA1*, *MACC1-AS1* and *LOC730101* to participate in the regulatory of AMPK signaling. Another group of lncRNAs, such as *HAND2-AS1* and *HOXC-AS3*, was upregulated under glucose starvation to modulate glycolysis. On the other hand, glucose deprivation induces *GAS5* and *Linc01564* to regulate metabolic plasticity in response to stress stimulation. *GIRGL*, *GLS-AS* and *UBA6-AS1* are shown to be glutamine deprivation-responsive lncRNAs, which regulates glutamine metabolism or maintains PARP1 function to determine cancer cell fate. ATF4 (activating transcription factor 4), PHGDH (phosphoglycerate dehydrogenase), MDH2 (malate dehydrogenase 2), FH (fumarate hydratase), CS (citrate synthase), LKB1 (liver kinase B1), AMPK (AMP-activated protein kinase), MACC1 (metastasis-associated in colon cancer protein 1), SP-1 (specificity protein 1), SIRT6 (sirtuin 6), HIF1α (hypoxia inducible factor 1 subunit alpha), PKM2 (pyruvate kinase M2), GRP78 (glucose-regulated protein), IDH1 (isocitrate dehydrogenase 1), FOXO (forkhead box O3), GLS (glutaminase), GCN2 (general control nonderepressible 2) and PARP1 (poly(ADP-ribose) polymerase 1). This figure was created with BioRender.com.

Cancer cells are featured of their high demand of nutrients since they have to proliferate and grow rapidly. Nonetheless, the limited vascularization in the core of a solid tumor often results in the deficiency of nutrients in the tumor microenvironment [88,89]. The inability of consuming enough nutrients due to the insufficient nutrient supply or the high demand of nutrients causes stress to the cancer cells, which is denoted as metabolic stress [90]. During tumor development, cancer cells often undergo metabolic reprogramming in order to support their rapid growth or survive from the metabolic stress [91]. Although a number of studies have already indicated the role of lncRNAs in regulating metabolic reprogramming [92] and cancer metabolism [93,94], here, we are focusing on how cancer cells respond or confront to metabolic stress through these nutrient deficiency-responsive lncRNAs (Figure 4).

As glucose is the major source of nutrient for most cancer cells, a significant portion of studies pays attention to how lncRNAs participate in metabolic stress response under glucose deficiency. AMP-activated protein kinase (AMPK) signaling is an important pathway activated by low glucose availability or nutrient deficiency [95] to regulate the growth, differentiation or metabolism of cancer cells in response to metabolic stress [96], and there are several lncRNAs reported to be induced upon glucose deprivation to participate in the AMPK signaling pathway. LncRNA *LOC730101* is upregulated by AMPK activation to promote cell survival and to inhibit apoptosis under glucose deprivation in osteosarcoma [97]. Liver kinase B1 (LKB1) is a kinase that activates AMPK through phosphorylation [98]. This LKB1/AMPK axis was found to induce lncRNA *MITA1* by promoting the DNA methylation of *MITA1* under glucose deficiency, and the upregulation of *MITA1* can further increase *SNAI2*/Slug expression to promote the metastasis of HCC cells [99]. In addition to be induced by AMPK signaling, another two lncRNAs are reported to activate AMPK under metabolic stress. *NBR2* is a lncRNA induced by LKB1-AMPK axis under glucose deprivation, and it can, in turn, bind to AMPK and promotes AMPK activity to form a feed-forward loop. The activation of AMPK signaling under this circumstance inhibits cell cycle progression and autophagy, resulting in reduced tumor growth [100]. Glucose starvation upregulates lncRNA *MACC1-AS1* to activate the AMPK/Lin28 axis, which improves mRNA stability and the protein expression of MACC1. The upregulation of MACC1 mediated by *MACC1-AS1* finally triggers MACC1-mediated metabolic plasticity, which enhances glycolysis and antioxidant capacity to promote tumor progression of GC [101].

Moreover, lncRNAs are upregulated under glucose starvation to regulate glycolysis through multiple mechanisms. The upregulation of lncRNA *TP53TG1* by glucose deprivation increases the mRNA expression of *GRP78* and *IDH1* and inhibits *PKM2* expression, which may contribute to a decrease in glycolysis. The alteration of these genes caused by *TP53TG1* further promotes cell growth and migration in glioma cells [102]. LncRNA *FILNC1* is upregulated by transcription factor FOXO under glucose deprivation for interacting with AU-binding factor 1 (AUF1), which prevents the binding of AUF1 on the *MYC*/c-Myc promoter and finally leads to a reduction in c-Myc expression. c-Myc is responsible for upregulating genes involved in glycolysis [103], so the downregulation of c-Myc caused by *FILNC1* inhibits glycolysis and ultimately represses renal tumor growth [104]. In osteosarcoma, glucose deficiency enhances the expression of lncRNA *HAND2-AS1*, which binds to Fructose-1, 6-bisphosphatase 1 (FBP1) to sequester the interaction between FBP1 and *HIF1α* mRNA, thereby influencing glycolysis-related genes [105]. The loss of FBP1 binding mediated by *HAND2-AS1* leads to a decrease in HIF1α translation, which attenuates glycolysis and further reduces tumor growth [106]. Finally, lncRNA *HOXC-AS3* is induced by transcription factor SP1 under glucose starvation. *HOXC-AS3* complexes with Sirtuin 6 (SIRT6), a histone deacetylase, to antagonize SIRT6 function, which results in the increase in HIF1α activity and the expression of glycolysis-related genes. This *HOXC-AS3*-mediated metabolic reprogramming eventually assists BC cells in adapting to metabolic stress [107]. In addition to glycolysis, lncRNAs also regulate other metabolic pathways when glucose is deficient. LncRNA *GAS5* is upregulated to promote the binding of malate dehydrogenase (MDH2) and histone deacetylase SIRT3 under glucose deprivation, which further results in a decrease in MDH2 acetylation. The reduction in acetylation loosens the interaction between fumarase (FH)-MDH2-citrate synthase (CS) complex, which reduces TCA flux and finally limits breast tumor growth [108]. Activating transcription factor 4 (ATF4) mediates the upregulation of lncRNA *Linc01564* under glucose starvation. *Linc01564* works as a miRNA sponge to sponge *PHGDH*-targeted miR-107 and miR-103a-3p, which increases the expression of PHGDH and further enhances serine synthesis pathway. More glutathione is produced by the enhanced serine synthesis pathway to relieve oxidative stress, helping cells survive from glucose starvation-induced metabolic stress [109].

Apart from glucose, cancer cells require amino acids, especially glutamine, to support their rapid growth. As a result, amino acid deficiency is also a common phenomenon observed during tumor development [89]. LncRNA *GLS-AS* is downregulated by c-Myc under glutamine deprivation. Under normal conditions, *GLS-AS* inhibits glutaminase (GLS) expression through Dicer-dependent RNA silencing, so the decrease in *GLS-AS* increases GLS expression under glutamine deficiency to confront with metabolic stress. In addition to helping cells survive from stress, the upregulation of GLS reciprocally maintains c-Myc stability to inhibit *GLS-AS* expression, which reinforces GLS-mediated cell survival under stress in pancreatic cancer [110]. Glutamine deprivation activates transcription factor c-Jun to upregulate lncRNA *GIRGL*, which inhibits cell proliferation under metabolic stress in colon cancer cells. Mechanically, *GIRGL* promotes the interaction between CAPRIN1 and *GLS1* mRNA, resulting in the inhibition of GLS1 translation and GLS1-mediated glutamine metabolism to inhibit cell growth [111]. Finally, an amino acid restriction-induced lncRNA *UBA6-AS1* is reported to help cells adapt to metabolic stress in BC cells. *UBA6-AS1* is upregulated through the GCN2/ATF4 axis to maintain PARP1 expression and function, which ultimately leads to a trade-off between migratory ability and cell survival under glutamine or arginine deprivation [112].

## 6. ER Stress

ER is an organelle responsible for calcium storage, lipid metabolism, protein synthesis and protein folding [113]. When cells encounter stress stimuli or ER dysfunction, a portion of proteins become misfolded or unfolded, and the aberrant accumulation of these misfolded or unfolded proteins in the ER leads to the occurrence of ER stress and further activates ER stress response, which is the so-called unfolded protein response (UPR) [114]. The activation of UPR attenuates protein synthesis or induces autophagy to recover cell homeostasis or triggers cell death in response to stress stimulation [115].

The UPR is initiated by three main ER transmembrane protein sensors: protein kinase-like ER kinase (PERK), inositol-requiring transmembrane kinase endoribonuclease-1α (IRE1α) and activating transcription factor 6 (ATF6). These sensors are bound by binding Ig protein (BiP), also known as GRP78, under homeostatic conditions to suppress their activation. As the misfolded or unfolded peptides accumulate in the ER lumen, BiP will in turn bind to the unfolded peptides and further release UPR sensors for activation to initiate the stress response cascades [116]. PERK is a kinase that phosphorylates eukaryotic translation initiation factor 2 subunit-α (eIF2α) at serine 51, which results in the attenuation of global protein synthesis but selectively increases the translation of an important stress-responsive transcription factor, ATF4. ATF4 then plays a dual role in promoting either cell survival or cell death depending on the types or duration of stress [117]. IRE1α is a ribonuclease activated by the unfolded protein aggregates to splice *X-box binding protein 1* (*XBP1*) mRNA to generate the functional transcription factor, spliced XBP1 (XBP1s). XBP1s can promote the transcription of genes that participate in protein folding and ER-associated degradation (ERAD) to eliminate the unfolded proteins and further relieve ER stress [118]. ATF6 is a transmembrane transcription factor that is retained on ER membranes by BiP under normal condition. Once released by BiP, ATF6 is transported to Golgi apparatus and cleaved by peptidases to generate an active transcription factor, cleaved ATF6α [119]. Cleaved ATF6α enters into the nucleus to promote transcription of XBP1 as well as genes involved in protein folding and ERAD, which helps resolve ER stress [120].

**Figure 5:**
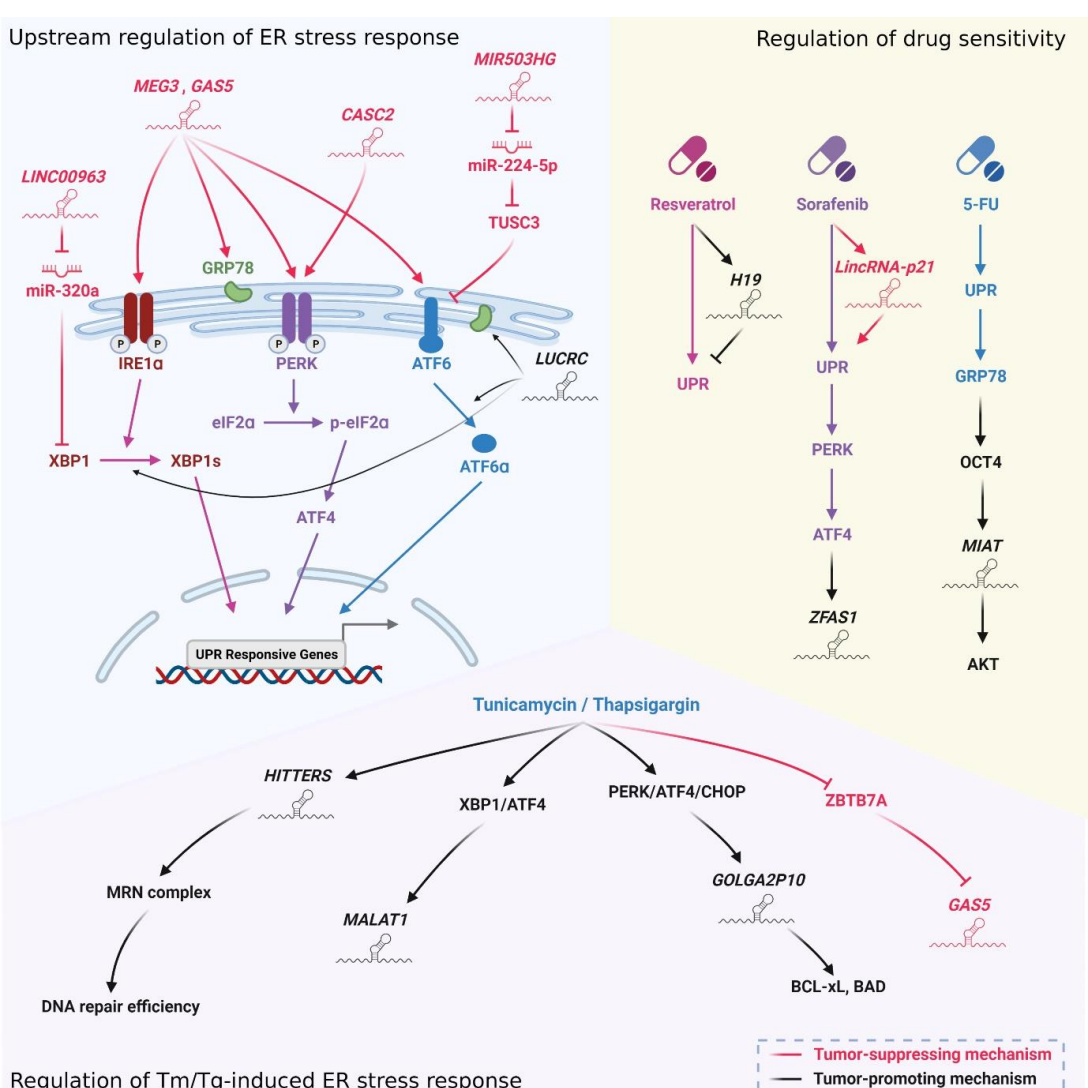
LncRNAs regulate the activation of ER stress response and the drug sensitivity to ER stress-inducing therapeutic agents. LncRNA *LINC00963*, *MEG3*, *GAS5*, *CASC2*, *MIR503HG* and *LUCRC* directly modulate UPR signaling to manage cell fate. Several chemotherapeutic drugs such as resveratrol, sorafenib and 5-FU increase lncRNA *H19* and *LincRNA-p21* expression to regulate UPR. In addition, these agents promote UPR signaling to upregulate *ZFAS1* and *MIAT* and further affect drug sensitivity. A subset of lncRNAs, including *HITTERS*, *MALAT1*, *GOLGA2P10* and *GAS5*, was induced by Tm/Tg-induced UPR to regulate tumor growth. XBP1 (X-box binding protein 1), IRE1α (inositol-requiring transmembrane kinase endoribonuclease-1α), GRP78 (glucose-regulated protein), PERK (protein kinase-like ER kinase), eIF2α (eukaryotic translation initiation factor 2 subunit-α), ATF4 (activating transcription factor 4), ATF6 (activating transcription factor 6), TUSC3 (tumor suppressor candidate 3), UPR (unfolded protein response), MRN complex (MRE11–RAD50–NBS1 complex), CHOP (C/EBP homologous protein), BCL-xL (B-cell lymphoma-extra large), BAD (BCL2 associated agonist of cell death) and ZBTB7A (zinc finger and BTB domain containing 7A). This figure was created with BioRender.com.

In multiple cancers, lncRNAs have been demonstrated to participate the UPR cascades in different aspects (Figure 5). Some lncRNAs are involved in the initiation of UPR by regulating the expression or the activation of UPR sensors. The overexpression of lncRNA *MEG3* [121,122] and *GAS5* [123] increases the expression of GRP78, IRE1, PERK, and ATF6, which may further trigger apoptosis to suppress tumor growth. *LUCRC*, highly expressed in colorectal tumor tissue to promote proliferation, migration and invasion of tumor cells, is required for the expression of BiP and the cleavage of *XBP1* and ATF6 to evoke UPR [124]. *CASC2* increases PERK mRNA stability to trigger the PERK/pS51-eIF2α/CHOP (C/EBP homologous protein) axis, which induces apoptosis in irradiated NSCLC cells [125]. *MIR503HG*, a miRNA sponge, increases *TUSC3* expression by sponging miR-224-5p. The increase in TUSC3 further inhibits ATF6-mediated UPR and decreases cancer cell viability, proliferation and epithelial–mesenchymal transition (EMT) [126]. *LINC00963* can sponge miR-320a, which directly targets *XBP1* 3′-UTR to abrogate *XBP1* expression. As a result, when *LINC00963* sponges miR-320a, XBP1-mediated UPR enhances apoptosis in GC [127]. On the other hand, ER stress can act as a stimulator of some lncRNAs. *GAS5* is a lncRNA that is suppressed by ZBTB7A under normal conditions in osteosarcoma cells. When the UPR is activated by ER stress inducers, tunicamycin (Tm) and thapsigargin (Tg), ZBTB7A is downregulated and results in the increase in *GAS5* to promote apoptosis [128]. *MALAT1*, one of the most well-characterized lncRNAs, is found to be induced by Tg and may be regulated by XBP1 and ATF4 to increase cell migratory abilities under ER stress in colorectal cancer cells [129]. *GOLGA2P10* is found to be increased in HCC and is regarded as an oncogenic lncRNA. Under Tm or Tg treatment, *GOLGA2P10* is upregulated by the PERK/ATF4/CHOP axis to increase BCL-xL expression and BAD phosphorylation, which decreases ER stress-induced apoptosis to assist cells in confronting ER stress [130]. LncRNA *HITTERS*, which is also upregulated by Tm or Tg, functions as an RNA scaffold to promote the interaction of the MRN complex. As ER stress can trigger DNA damage and may further induce apoptosis, the increase in MRN complex interactions can maintain DNA repair efficacy to prevent ER stress-induced apoptosis, which suggests that *HITTERS* is a pro-survival molecule in UPR [131]. In addition to participating in the regulation of UPR, some lncRNAs have been shown to either sensitize or counteract the efficacy of ER stress-inducing chemotherapeutic or targeted therapeutic agents. A potent anti-cancer agent resveratrol initiates UPR and enhances the expression of lncRNA *H19*. The combination of resveratrol and knockdown of *H19* increases the expression of ER stress-related genes, promotes cell death and decreases EMT-related genes, suggesting the role of *H19* in resveratrol-resistant GC cells [132]. Sorafenib, as a first line protein kinase inhibitor for treating advanced HCC [133], can induce UPR signaling to trigger apoptotic cell death with the assistance of *lincRNA-p21* [134]. This study indicates that *lincRNA-p21* is a tumor suppressive lncRNA in HCC and contributes to the activation of UPR signaling and apoptosis under sorafenib treatment. Another lncRNA *ZFAS1* is shown to be upregulated by PERK/ATF4 axis after sorafenib treatment in sorafenib-resistant HCC cells, which suggests that *ZFAS1* may be a therapeutic or prognostic biomarker of sorafenib treatment in HCC patients [135]. 5-FU is one of the most commonly used anti-cancer drugs which is mis-incorporated into RNA and DNA and further leads to cell death [136]. In 5-FU resistant cells, lncRNA *MIAT* is found to be upregulated. Mechanically, 5-FU evokes UPR and upregulates *MIAT* through the GRP78/octamer-binding transcription factor 4 (OCT4) pathway to confer 5-FU resistance in BC cells [137].

## 7. Stress-Responsive lncRNAs as Emerging Biomarkers for Assessment of Prognosis and Prediction of Therapy Response in Cancer

During cancer progression and therapy, cellular stress responses have been designated to regulate cell fate decision and susceptibility to therapeutic agents. It is an emerging fact that lncRNAs play indispensable roles in these processes. LncRNAs participate in not only the initiation or activation of stress sensors but also the regulation of biological pathways in response to various stress stimulations. In this review, we summarized the regulatory role of lncRNAs and emphasized the molecular mechanisms of how lncRNAs participate in the five major stress conditions including genotoxic, hypoxic, oxidative, metabolic and ER stress. The involvements of lncRNAs in the stress response in cancer and their associations with tumor progression and the effectiveness of cancer therapeutics are highlighted (Table 1). We then propose that these stress-responsive lncRNAs are ideal for being developed into cancer biomarkers for assessing disease prognosis and predicting therapy response.

While a significant amount of studies has reported lncRNAs that are either upregulated or downregulated in various types of cancer compared to normal tissues [21,138], the identification of stress-responsive lncRNAs in cancer reveals a new layer of regulation and paves the way for biomarker development. The expression of *lincRNA-p21* is deregulated in many cancers including skin, prostate, colorectal cancer and chronic lymphocytic leukemia [139], and it has been proposed to serve as a marker for diagnosing cancer. Moreover, *lincRNA-p21* has also been found to play diverse roles in genotoxic, hypoxic and ER stress responses during cancer progression [29,54,134], indicating that when combining with specific stress-responsive factors, *lincRNA-p21* has the potential to serve as a biomarker for precisely predicting disease prognosis or therapy response. *NEAT1* participates in cancer progression and therapeutic response under various conditions, and it seems to be an ideal prognostic marker for assessing hypoxic tumors due to its tumor-promoting role under hypoxia [58,65]. Moreover, *NEAT1* may also become a promising biomarker when assessed in combination with other markers. Based on the studies demonstrating the roles of *NEAT1* in regulating cancer response to the ferroptosis inducer, erastin [83,84], it is plausible that by the co-evaluation of *NEAT1* and miR-362-3p expression, one can predict the tumor response to erastin.

Many cancer therapeutic drugs, such as oxaliplatin and As_2_O_3_ and erastin, 5-FU and TMZ abrogate tumor growth by inducing stress, suggesting that potential therapeutic targets can be developed for reversing the lncRNA-mediated resistance of these drugs. In addition, lncRNAs associated with the resistance to these cancer therapeutic drugs, for example, *BORG*, *MALAT1*, *SBF2-AS1*, *HIF1A-AS1*, *LINC01134*, *NEAT1*, *LAMTOR5-AS1*, *NORAD*, *lincRNA-p21*, *ZFAS1* and *MIAT*, may also serve as promising biomarkers for the diagnosis of therapy-resistant cancers or the prediction of therapy outcome. *SBF2-AS1* and *MALAT1* are associated with TMZ-resistant GBM [41,42]. Specifically, *SBF2-AS1* is highly present in the exosomes secreted by TMZ-resistant GBM cells, suggesting that the detection of serum *SBF2-AS1* holds hope for diagnosing therapy-resistant GBM [42]. Hypoxic renal cancer cells secrete exosomes containing *lncHILAR* to promote cell invasion and migration [62]. Therefore, serum *lncHILAR* can be a biomarker to evaluate disease progression and patient outcome. In chemo-resistant gastrointestinal cancers, *LINC01134* [77], *NORAD* [78] and *HIF1A-AS1* [55] are all elevated and could be used for predicting responsiveness to chemotherapies. *BORG* [40] and *MIAT* [137] are both associated with the chemoresistance of BC and may reflect the clinical outcomes of patients treated with a commonly used chemotherapy drug, 5-FU. Finally, cancer metabolism has gradually been regarded as potential therapeutic targets [140] or the causes of therapeutic resistance [141] in cancer treatments. Since several lncRNAs are found to regulate cell survival under metabolic stress, potential therapeutic strategies or prognostic markers can be developed based on these studies. AMPK is a promising target for cancer treatment, and therapeutic strategies have been developed for activating or inhibiting AMPK [142]. There are several lncRNAs including *NBR2*, *MITA1*, *LOC730101* and *MACC1-AS1* associated with AMPK activity and may serve to assess the response of AMPK modulation in cancer. The inhibition of GLS has also been proposed for suppressing tumor growth and metastasis [140]. *GIRGL* and *GLS-AS* are reported to inhibit GLS expression to impede tumor progression [110,111], suggesting that these lncRNAs have the potential to become therapeutic targets. *UBA6-AS1* participates in the amino acid deficiency-induced integrated stress response and regulates the activity of PARP1 for BC cell survival. The level of *UBA6-AS1* correlates with the cellular response to PARP inhibitors [112], implying that *UBA6-AS1* may be considered as a biomarker for predicting breast tumor response to PARP inhibitors.

Taken together, increasing number of studies has uncovered and recognized the importance of lncRNAs in the regulation of cellular stress response during cancer progression and therapy. It would be worthwhile to further investigate and explore the possibility of developing these stress-responsive lncRNAs into biomarkers that can be applied clinically for assisting disease stratification in the future.

## Figures and Tables

**Table 1:** List of stress-responsive lncRNAs associated with cancer progression and therapy resistance.

Type of Stress	lncRNA	Type of Cancer	Function (Tumor Promoter/ Suppressor)	Therapy Resistance	Reference
Genotoxic stress	*BORG*	Breast cancer	Promoter	Doxorubicin	[40]
	*DlincRNA*	-	Promoter	-	[36]
	*ERIC*	-	Promoter	-	[34]
	*HITT*	-	Suppressor	-	[38]
	*HOTAIR*	Ovarian cancer	Promoter	Platinum-based drugs	[35]
	*LincRNA-p21*	Skin cancer	Suppressor	-	[29]
	*LINP1*	Breast cancer	Promoter	-	[37]
	*MALAT*	Glioblastoma	Promoter	Temozolomide	[41]
	*NBAT1*	Neuroblastoma	Suppressor	Cisplatin Doxorubicin Etoposide	[31]
	*Nrf2-lncRNA*	Hepatocellular carcinoma	Promoter/Suppressor	Doxorubicin	[43]
	*Pvt1b*	Lung cancer	Suppressor	-	[33]
	*SBF2-AS1*	Glioblastoma	Promoter	Temozolomide	[42]
	*SPARCLE*	-	Suppressor	Doxorubicin	[32]
Hypoxic stress	*AC020978*	Lung cancer	Promoter	-	[53]
	*BCRT*	Breast cancer	Promoter	-	[59]
	*BX111*	Pancreatic cancer	Promoter	-	[51]
	*CF129*	Pancreatic cancer	Promoter	-	[63]
	*HIF1A-AS1*	Pancreatic cancer	Promoter	Gemcitabine	[55]
	*HILAR*	Renal cancer	Promoter	-	[62]
	*LHFPL3-AS2*	Lung cancer	Promoter	-	[66]
	*LincRNA-LET*	Hepatocellular carcinoma Lung cancer Colorectal cancer	Promoter	-	[64]
	*LincRNA-p21*	-	Promoter	-	[54]
	*NEAT1*	Breast cancer	Promoter	-	[65]
		Hepatocellular carcinoma	Promoter	-	[58]
	*PCGEM*	Gastric cancer	Promoter	-	[50]
Oxidative stress	*GABPB1-AS1*	Hepatocellular carcinoma	Suppressor	Erastin	[85]
	*GAS5*	Melanoma	Suppressor	-	[80]
	*H19*	Cholangiocarcinoma	Promoter	-	[72]
	*HULC*	Cholangiocarcinoma	Promoter	-	[72]
	*LAMTOR5-AS1*	Osteosarcoma	Suppressor	Etoposide Carboplatin Cisplatin	[70]
	*LINC00336*	Lung cancer	Promoter	-	[86]
	*LINC01134*	Hepatocellular carcinoma	Promoter	Oxaliplatin	[77]
	*NEAT1*	Hepatocellular carcinoma Non-small cell lung cancer	Promoter	Erastin	[84]
	*NORAD*	Gastric cancer	Promoter	Oxaliplatin	[78]
	*OIP5-AS1*	Prostate cancer	Promoter	Cadmium	[87]
	*OTUD6B-AS1*	Bladder cancer	Suppressor	As_2_O_3_	[71]
	*ROR*	Hepatocellular carcinoma	Promoter	As_2_O_3_	[74]
	*SCAMP1*	Renal cell carcinoma	Suppressor	-	[75]
	*THAP9-AS1*	Osteosarcoma	Promoter	-	[79]
	*XIST*	Osteosarcoma	Promoter	-	[73]
Metabolic stress	*FILNC1*	Renal cell carcinoma	Suppressor	-	[104]
	*GAS5*	Breast cancer	Suppressor	-	[108]
	*GIRGL*	Colon cancer	Suppressor	-	[111]
	*GLS-AS*	Pancreatic cancer	Promoter	-	[110]
	*HAND2-AS1*	Osteosarcoma	Suppressor	-	[106]
	*HOXC-AS3*	Breast cancer	Promoter	-	[107]
	*Linc01564*	Hepatocellular carcinoma	Promoter	-	[109]
	*LOC730101*	Osteosarcoma	Promoter	-	[97]
	*MACC1-AS1*	Gastric cancer	Promoter	-	[101]
	*MITA1*	Hepatocellular carcinoma	Promoter	-	[99]
	*NBR2*	Breast cancer Renal cell carcinoma	Suppressor	-	[100]
	*TP53TG1*	Glioma	Promoter	-	[102]
	*UBA6-AS1*	Breast cancer	Promoter	-	[112]
ER stress	*CASC2*	Non-small cell lung cancer	Suppressor	-	[125]
	*GAS5*	Hepatocellular carcinoma	Suppressor	-	[123]
		Osteosarcoma		-	[128]
	*GOLGA2P10*	Hepatocellular carcinoma	Promoter	-	[130]
	*H19*	Gastric cancer	Promoter	Resveratrol	[132]
	*HITTERS*	Oral squamous cell carcinoma	Promoter	-	[131]
	*LINC00963*	Gastric cancer	Suppressor	-	[127]
	*LincRNA-p21*	Hepatocellular carcinoma	Suppressor	Sorafenib	[134]
	*LUCRC*	Colon cancer	Promoter	-	[124]
	*MALAT1*	Colon cancer	Promoter	-	[129]
	*MEG3*	Breast cancer	Suppressor	-	[121]
		Hepatocellular carcinoma		-	[122]
	*MIAT*	Breast cancer	Promoter	5-FU	[137]
	*MIR503HG*	Gastric cancer	Suppressor	-	[126]
	*ZFAS1*	Hepatocellular carcinoma	Promoter	Sorafenib	[135]
